# Involvement of a universal amino acid synthesis impediment in cytoplasmic male sterility in pepper

**DOI:** 10.1038/srep23357

**Published:** 2016-03-18

**Authors:** Xianping Fang, Hong-Fei Fu, Zhen-Hui Gong, Wei-Guo Chai

**Affiliations:** 1 Institute of Biology, Hangzhou Academy of Agricultural Sciences, Hangzhou 310024, China; 2 Institute of Vegetables, Hangzhou Academy of Agricultural Sciences, Hangzhou 310024, China; 3 College of Horticulture, Northwest A&F University, Yangling 712100, China

## Abstract

To explore the mechanisms of pepper (*Capsicum annuum* L.) cytoplasmic male sterility (CMS), we studied the different maturation processes of sterile and fertile pepper anthers. A paraffin section analysis of the sterile anthers indicated an abnormality of the tapetal layer and an over-vacuolization of the cells. The quantitative proteomics results showed that the expression of histidinol dehydrogenase (HDH), dihydroxy-acid dehydratase (DAD), aspartate aminotransferase (ATAAT), cysteine synthase (CS), delta-1-pyrroline-5-carboxylate synthase (P5CS), and glutamate synthetase (GS) in the amino acid synthesis pathway decreased by more than 1.5-fold. Furthermore, the mRNA and protein expression levels of DAD, ATAAT, CS and P5CS showed a 2- to 16-fold increase in the maintainer line anthers. We also found that most of the amino acid content levels decreased to varying degrees during the anther tapetum period of the sterile line, whereas these levels increased in the maintainer line. The results of our study indicate that during pepper anther development, changes in amino acid synthesis are significant and accompany abnormal tapetum maturity, which is most likely an important cause of male sterility in pepper.

Cytoplasmic male sterility (CMS) is a natural phenomenon that widely occurs in plants that express maternal inheritance, pollen abortion and normal pistils and have the ability to restore fertility by dominant restoring genes^1^. International hybrid seed production commonly uses CMS to breed sterile line hybrid seeds because the use of this process allows for the omission of artificial emasculation, conserves manpower and material resources, increases the purity of hybrid seeds and increases the output of crops. However, in practical seed selection processes, a number of problems occurs, such as cytoplasm singularity, low combining ability and unstable sterility, and farmers may lack the theoretical knowledge required to solve these problems. The CMS line is an ideal material in the study of nucleo-cytoplasmic interactions; thus, scientists have reported numerous studies on the mechanisms of sterility^2^,^3^,^4^,^5^,^6^,^7^,^8^,^9^,^10^,^11^,^12^ and identified many CMS-related genes (orf507^2^,^8^, atp6^10^ and orf456^11^ in pepper; AcPMS1 in onion^4^; and MYB80/UNDEAD in Arabidopsis^7^, among others) and proteins (ATP synthase and Hsp60 in wheat^3^). Because CMS widely occurs, plant pollen abortion has different symptoms, although its common morphological expression is as small floral organs, short filaments, and thin and small anthers. The microspore development of the anthers usually begins with stages of pollen mother cells (stage I) and the tetrad period of meiosis (stage II). In the CMS line, the tapetal cells begin to disintegrate (stage III) and become highly vacuolized (stage IV) in the cytological structure, and then the pollen mother cells disintegrate (stage V), thereby causing degeneration. In the maintainer line, however, stage III is a dual-core stage, after which pollen grains form (stage IV) and escape from the pollen sacs (stage V). CMS can be classified into two types according to the period during which pollen abortion occurs^13^,^14^: sporophyte sterility and gametophyte sterility. The tapetal layer is composed of a special type of secretory cell that plays an important role in the formation of pollen. In the early period of anther development, the tapetal layer surrounds the anther to provide a variety of nutritive substances to the developing microspores. After the blast cells of the microspore have undergone meiosis, the tapetal layer will secrete a callose enzyme (β-1,3-glucanase) that decomposes the callose wall of the cyst tetraspore to release microspores^15^. Both the early decomposition and delayed down-regulation of this enzyme most likely lead to an insufficient supply of nutrition to the spores or prevent normal separation, thus causing pollen abortion^16^,^17^. Therefore, it is important to explore the cause of the abnormal development of the tapetal-layer cells and study the CMS molecular mechanisms using comprehensive biochemical and molecular biological methods.

With continuous intensive research and the development of novel technologies, proteomics has become a hot spot of contemporary life science research. When researching plant genetic functions and molecular mechanisms using proteomics technology, it is of great importance to identify plant genes and explore changes in major physiological signal channels. Proteomics has become a powerful tool with which to study the physiological and biochemical mechanisms of field crops^18^,^19^ and has been widely used in molecular mechanism research on rice^20^,^21^,^22^, wheat^3^,^23^, strawberry^24^,^25^, rape^26^, soybean^27^ and other crops^18^. Furthermore, proteomics has also been used to explore the CMS mechanisms of various plants, such as pepper^28^,^29^, rice^30^, tomato^31^, *Brassica napus*^32^, wolfberry^33^, ad tobacco^34^. Zhang *et al.*^28^ found 13 proteins that were detected differently between the pepper CMS line and its maintainer line using two-dimensional difference gel electrophoresis (2D-DIGE), and Wu *et al.*^29^ found 23 proteins with a greater than 1.5-fold change in either the pepper CMS or its maintainer by two-dimensional gel electrophoresis (2DE). However, compared with the results obtained through 2DE/DIGE, we will obtain better high-throughput and reproducible results using liquid chromatography-tandem mass spectrometry (LC-MS/MS). Although this process is labour intensive, investigating proteome changes during pepper anther development will provide meaningful data for further explorations of pepper CMS mechanisms.

Because free amino acids account for 6% of the dry weight of plant pollen^35^, the levels of free amino acids may be closely related to pollen production and, therefore, anther development. Many scientists have studied the changes in free amino acid content during anther development in fertile and CMS plants. Wu *et al.*^36^ found 2- to 3-fold and 10- to 20-fold higher levels of proline and pipecolic acid, respectively, in the fertile line compared with the CMS line of Petunia. Rai’s study of wheat^37^ revealed that among the bound amino acids, proline was lower and aspartic and glutamic acid were higher in male sterile anthers than in fertile anthers. Mattioli^38^ also reported that proline is required for male gametophyte development in Arabidopsis. Nevertheless, few scientists have systematically studied the expression changes of 17 common types of amino acids in CMS pepper, which is one of the objectives of this study.

Our research group used two pepper CMS samples that were imported from the Centre for Genetic Resources, the Netherlands, and after four years of directional separation and purification, we have established both a stable pepper CMS line (DH-01-1-1A) and its maintainer line (DH-01-1-1B). In this research, we used paraffin selections to observe the cytobiological structural changes of the anthers of DH-01-1-1A and DH-01-1-1B during each of their developmental stages and identified the differentially expressed proteins qualitatively and quantitatively based on high-resolution mass spectrum proteomic methods. We observed changes in the major metabolic pathways during the anther tapetal layer stage between the CMS line and the maintainer line and have further reported the relevant mechanisms of pepper CMS, which will help elucidate pepper CMS for use in heterosis breeding.

## Materials and Methods

### Experimental materials

The stable pepper CMS line (DH-01-1-1A) and its maintainer line (DH-01-1-1B) were developed by the Institute of Vegetables of Hangzhou Academy of Agricultural Sciences, Zhejiang, China. Our research group introduced the hot pepper cultivar DH-01-1 from the Netherlands in 2009. The cultivar DH-01-1 presented a separation of fertility in the second generation. We chose the male sterile plant as the mother line and matched 0412-2 as the recurrent parent, which had excellent integrated characters. After five backcrosses, the stable male sterility line DH-01-1-1A (100% sterile, not sensitive to temperature or light) with good integrated characters was produced and selected for the experiments. The pepper materials that were used in the experiments were bred during the first ten-day period of December 2013 and then transplanted to the greenhouse on March 3, 2014. In the first ten days of June 2014, the pepper materials began their florescence period, and from 8–9 AM, we began to select buds at five different developmental stages. Subsequently, we stripped the anthers in the buds with tweezers for later use.

### Paraffin sections of the anthers

The anthers were immersed directly in Carnoy’s fixative solution containing ethanol, chloroform, and acetic acid (6:3:1). After 24 h, the anthers were collected and dehydrated through an ethanol series of 70, 85, 95 and 100% (v/v), and xylene was used to induce transparency. Buds of different sizes were picked, and then routine paraffin section procedures were used to embed the buds, which were cut into serial sections of 10 μm in thickness. The anthers were dyed and mounted by hematoxylin and neutral balsam, respectively. Photographs and records were collected using an electron microscope (Leica DM1000, Germany). Approximately 10 g of anthers was collected according to the five different developmental stages of the microspores for use in later experiments.

### Protein extraction and tryptic digestion

A portion (2 g) of fresh sterile line pepper anther was first ground in liquid nitrogen using a mortar and pestle. The powder was then transferred to a 50 mL centrifuge tube and precipitated with cold 10% TCA/acetone supplemented with 50 mM DTT, 0.1% Protease Inhibitor Cocktail Set VI and PVPP powder for 2 h at −20 °C. After centrifugation at 20,000 × *g* at 4 °C for 10 min, the supernatant was discarded. The remaining precipitate was washed three times with cold acetone supplemented with 50 mM DTT and 1 mM PMSF. After air drying, the precipitate was resuspended in lysis buffer (8 M urea, 2 mM EDTA, 10 mM DTT and 0.1% Protease Inhibitor Cocktail Set VI). The sample was sonicated three times on ice using a high-intensity ultrasonic processor (Scientz). The remaining debris was removed by centrifugation at 20,000 × *g* at 4 °C for 10 min. The supernatant was transferred to a new tube, and the proteins were reduced with 10 mM DTT for 1 h at 56 °C and alkylated with 55 mM iodoacetamide for 45 min at room temperature in the dark. The proteins were precipitated with 3 volumes of pre-chilled acetone for 30 min at −20 °C. After centrifugation, the pellet was then dissolved in 0.5 M TEAB and sonicated for 5 min. Following a second centrifugation step as above, the supernatant was collected. The protein concentrations were determined using a Bio-Rad Protein Assay Kit (standard Bradford method) with BSA as the calibration standard^39^. Approximately 100 μg of protein for each replicate was digested with trypsin (Promega) overnight at 37 °C in a 1:50 trypsin-to-protein mass ratio. The resulting peptides were cleaned with C18 ZipTips (Millipore) according to the manufacturer’s instructions and then analyzed by liquid chromatography-mass spectrometry (LC-MS/MS).

### LC-MS/MS analysis and database search

The peptides were dissolved in 0.1% FA, directly loaded onto a reversed-phase column and eluted with a linear gradient of solvent B (0.1% FA in 98% ACN) on an EASY-nLC 1000 UPLC system as follows: 1–40%, 120 min; 40–65%, 5 min; 65–65%, 5 min; 65–1%, 1 min; and 1%, 20 min equilibrium. The constant flow rate was 200 nL/min. The resulting peptides were analyzed by a Q Exactive^TM^ Hybrid Quadrupole-Orbitrap mass spectrometer (Thermo Fisher Scientific).

The peptides were subjected to nano-spray ionization followed by MS/MS in Q Exactive coupled online with UPLC. Intact peptides were detected using an Orbitrap at a resolution of 70,000. The peptides were selected for MS/MS using 25% normalized collisional energy (NCE) with 12% stepped NCE, and the fragments produced from low, medium and high collisional energy were simultaneously combined and detected^40^. Ion fragments were detected using an Orbitrap at a resolution of 17,500. A data-dependent procedure that alternated between one MS scan followed by 20 MS/MS scans was applied for the top 20 precursor ions above a threshold ion count of 3E4 in the MS survey scan with 15.0 s dynamic exclusion. The electrospray voltage was 2.0 kV. Automatic gain control was used to prevent the overfilling of the ion trap, and 1E5 ions were accumulated for the generation of MS/MS spectra. For the MS scans, the m/z scan range was 350 to 1800.

Because pepper and *Arabidopsis thaliana* are both dicotyledonous plants and the pepper proteome sequence information is limited (approximately 1600 proteins in the UniProt database), we used the database of the dicotyledon model organism *A. thaliana* in the NCBI database (http://www.ncbi.nlm.nih.gov/protein, *A. thaliana*, 228,789 sequences) for homologous sequence alignment. We identified the proteins using Thermo Fisher Proteome Discoverer 1.4 as the sequence alignment software and comparatively quantified the proteins by comparing the spectrum peak strength of differential expression using the Thermo Fisher Sieve 2.2 software (P-value < 0.01).

### Bioinformatics analysis

The UniProt database (http://www.ebi.uniprot.org) was searched to determine the functions of the identified proteins. Three independent ontological sets were used to annotate and group the proteins according to biological process, molecular function, and cellular compartmentalization. MultiExperiment Viewer 4.3 software was used for a differential expression spectrum clustering analysis of the differential proteins. STRING 9.1 software was used to analyze the possible protein-protein interaction (PPI) networks. The Kyoto Encyclopaedia of Genes and Genomes (KEGG) web pathway database (http://www.genome.jp/kegg/pathway.html) was used to match the proteins that were identified as relevant to the development process and identify the major metabolic pathways that might change because of a change in the expression of differential proteins (P-value < 0.01).

### Quantification of gene expression through quantitative real-time PCR

To further understand the mRNA expression levels of the corresponding proteins that were screened by the proteomics methods, we selected six proteins (histidinol dehydrogenase (HDH), dihydroxy-acid dehydratase (DAD), aspartate aminotransferase (ATAAT), cysteine synthase (CS), delta-1-pyrroline-5-carboxylate synthase (P5CS), and glutamate synthetase (GS)) with significant differential expression in metabolic pathways and analyzed the mRNA expression levels of their corresponding genes. A BLASTp search of the NCBI database was used to compare and select the homologous sequence of every selected protein, and the software CODEHOP was used to design the degenerate primers (GDP) in the conserved region. The middle fragments of the cDNAs were amplified. Primer 5.0 was used to design the specific primers (GSP) for use in the 5′ and 3′ RACE amplifications. All of the primers were synthesized by Shengong Biological Engineering (Shanghai) Corporation, Ltd. (Supplementary Table S1).

The total RNA of the pepper anthers was extracted, and the first-chain cDNA was synthesized. The degenerate primers GDP-S and GDP-A of each gene were used to amplify the middle fragments of cDNA. The specific primers 5′ GSP and 3′ GSP were used in the RACE reactions. The PCR products were treated using cataphoresis, gel-slice purification, connection, transformation and sequencing, and DNAMAN 6.0 software was used to splice the full-length cDNA sequence of each gene. Based on the splice sequence, the full-length forward primer GFP-S and reverse primer GFP-A were designed, and the full-length cDNA sequence of each gene was PCR amplified.

The quantitative PCR primers GRTP-S and GRTP-A were designed according to the internal reference gene sequence and the full-length cDNA sequence of each selected gene. All of the primer information is listed in Supplemental Table S1. The total RNA was extracted from the anthers of the pepper CMS line and maintainer line at different developmental stages using TRIzol reagent according to the supplier’s recommendations (Invitrogen, Germany). Residual DNA was removed with RNase-free DNase (Fermentas, Canada). Then, 1 μg of total RNA was reverse transcribed using 0.5 μg of Oligo (dT) 20 and 200 units of ReverTra Ace (TOYOBO, Japan) following the suppliers’ recommendations. Quantitative real-time PCR was performed using the Opticon 2 Real-time PCR Detection System (Bio-Rad, USA). The PCR assay was performed using SYBR Green Supermix (Bio-Rad, USA). The PCR conditions consisted of 40 cycles of denaturation at 95 °C for 30 s, annealing at 58 °C for 45 s, and extension at 72 °C for 30 s. To verify that a single product had been amplified, a dissociation curve was generated at the end of each PCR cycle using the software that was provided with the Opticon 2 Real-time PCR Detection System. To minimize sample variations, the mRNA expression of each target gene was normalized to the expression of the housekeeping gene glyceraldehyde-3-phosphate dehydrogenase (GAPDH, gene symbol AJ246013). All of the experiments were repeated three times with cDNA from the three samples. The mRNA was quantified using the comparative threshold cycle (Ct) method^41^. The Ct value for GAPDH (the internal standard) was subtracted from that of the gene of interest to obtain a ΔCt value. The Ct value of the control sample (anthers at stage I) was subtracted from the ΔCt value to obtain a ΔΔCt value. Each ratio change in expression level relative to that of the control was expressed as 2−ΔΔCt.

### Preparation of antiserum and immunoblotting

According to the amino acid sequences of the selected proteins (HDH, DAD, ATAAT, CS, P5CS, GS, and GAPDH (reference protein)), the respective peptide fragments (63-CVNPIIDAVRSNGDN-76, 102-CMHIIKLSEAVKEGV-115, 121-CGINAIREGFTRYTL-134, 77-CDNAAQLIGKTPMVY-90, 47-CELNSDGFEVILVSS-60, 197-CVKWPLGWPVGAFPG-210, and 18-CHRSQASCVGLQHSS-31) were used as protein-surface antigens because of their favourable properties with respect to antigenicity, relative hydrophobicity/hydrophilicity, flexibility, secondary structure, and aggregation potential. The seven peptide fragments were derived from the conserved protein sequences of the UniProt accessions Q9C5U8 (HDH), Q9LIR4 (DAD), Q9SIE1 (ATAAT), P47999 (CS), P54887 (P5CS), Q9LV03 (GS), and CAC80377 (GAPDH). Each peptide, along with an additional N-terminal cysteine (not part of the protein sequence), was synthesized and purified on a resin. Each purified peptide was used to raise polyclonal antibodies in rabbits (Abmart Biotechnology Co., Ltd., China). Protein samples were extracted and purified according to the methods described in Section 2.3. The total protein (20 μg) from each sample was subjected to electrophoresis through a 15% (w/v) SDS-PAGE gel. The proteins in the gel were electrotransferred onto a PVDF membrane, which was blocked with 5% (w/v) skim milk. The blot was incubated with the rabbit antiserum raised against each corresponding protein diluted to 1:1000 in TBST [25 mM Tris base (pH 8.0), 140 mM NaCl, 3 mM KCl, and 0.05% (v/v) Tween 20] for 1 h and washed three times for 5 min each in TBST. The blot was then probed with secondary antibody [HRP-labelled goat anti-rabbit IgG (H+L)] diluted to 1:2500, and reactive bands were visualized using ECL (Multisciences Biotech Co., Ltd., China). Each experiment was performed in triplicate. The band intensities of the six proteins were normalized to that of GAPDH at each developmental stage.

### Content measurement of the 17 amino acids

To compare the changes in amino acid content in the different developmental stages of the CMS line and maintainer line, we further measured the content of the 17 common amino acids during anther development. Anthers (0.1 g) of different developmental stages were weighed in special glass tubes, 10 mL of 6 M HCl was added, and then the tubes were sealed after vacuum treatment. The tubes were placed in dry boxes at a constant temperature of 110 °C to hydrolyse for 24 h, after which they were allowed to cool. Then, the hydrolysate was diluted with distilled water to a constant volume of 50 mL, and 1 mL of this solution was dried in a vacuum and ground into a powder. The powder was then homogenized in 1.2 mL of 0.02 M HCl and filtered through a 0.22-μm filter paper to obtain the sample solution. The amino acid content was measured using a Hitachi L-8800 automatic amino acid analysis unit with an ion exchange resin separation column that had a diameter of 4.6 mm × 60 mm (ion exchange resin 2622SC). The temperature of the separation column was set at 57 °C. The flow speed of the buffer solution and ninhydrin was 0.4 mL/min (pressure 7.0–8.5 MPa) and 0.35 mL/min (pressure 0.9–1.1 MPa), respectively. The detection limit threshold value was 3 pmol. The standard amino acid sample (mix of 17 amino acids) was purchased from Sigma-Aldrich, and it contained 2.5 μmol/mL each alanine, arginine, aspartic acid, glutamic acid, glycine, histidine, isoleucine, leucine, lysine, methionine, phenylalanine, proline, serine, threonine, tyrosine and valine and 1.25 μmol/mL cystine. The sampling volume of the analyzed samples and standard samples was 20 μL, and each experiment was replicated three times.

## Results and Discussion

### Cytological observation of pepper anthers at different developmental stages

**Figure 1:**
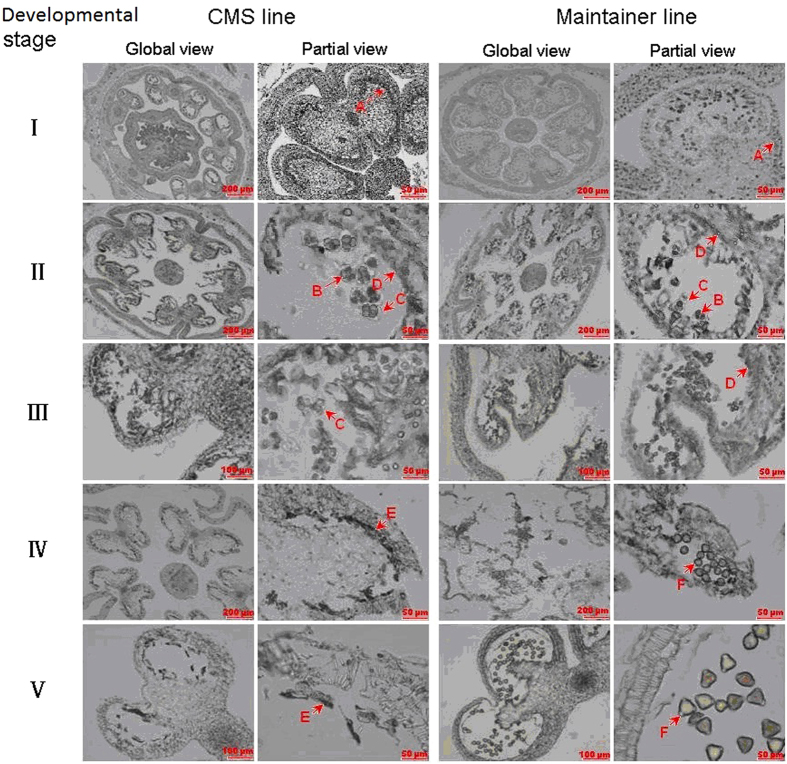
Microspore development of the pepper anther CMS line and the maintainer line. Development of microspores in the CMS line DH-01-1-1A: (**I**) pollen mother cells; (**II**) tetrad period of meiosis; (**III**) disintegration of tapetal cells; (**IV**) cell vacuolization; and (**V**) retrogression and disintegration of pollen mother cells. Development of microspores in the maintainer line DH-01-1-1B: (**I**) pollen mother cells; (**II**) tetrad period of meiosis; (**III**) dual-core stage; (**IV**) pollen grains; and (**V**) pollen loose powder. Different capital letters represent different cytological structure as follows: A, pollen mother cells; B, tetrad-stage cells; C, callose; D, tapetal cells; E, cell residues; and F, pollen grains.

To determine the cause of anther sterility, pepper anthers of the CMS line and maintainer line were selected at five different developmental stages and a paraffin section analysis was performed. Our results showed that in the pollen mother cell stage, the sterile and maintainer lines did not present significant difference in their cytological structure, whereas a significant difference was observed in the CMS line beginning in the tetrad period of meiosis, after which pollen abortion occurs. Because the callose around the tetrad cannot be decomposed and the tapetal layer cells are over-vacuolized, the tetrad gradually becomes hypertrophic in the radial direction, which squeezes it to the centre of the anther cell. The protoplast subsequently becomes dense, and then decomposition and pollen abortion occur gradually (Fig. 1).

Laser *et al.*^42^ reported that angiosperm CMS line pollen abortion may occur at various stages, although mostly at or close to the dual-core stage in monocots and the tetrad stage or early microspore stage in dicots. Through observations of paraffin sections, Luo *et al.*^12^ reported that the tapetum of pepper CMS 21A at the uninucleate stage swelled abnormally and was pressed against the pollen grains of the locule. Other results have also shown that the pepper CMS line begins to degrade after the tetrad stage^43^,^44^, which is consistent with our results.

### Proteome change in the anthers of the pepper CMS line at different developmental stages

**Figure 2:**
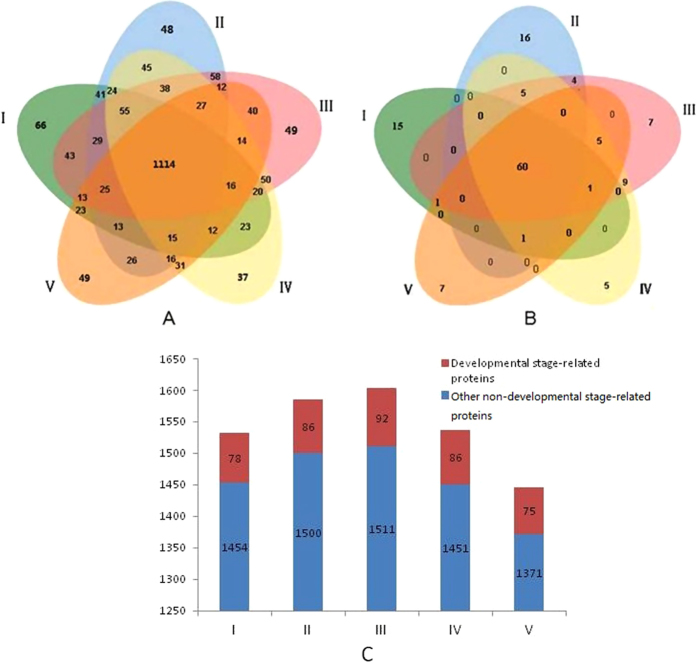
Summary charts of the identified proteins in pepper anthers (CMS line). Venn diagram of the total proteins (**A**) and the 136 developmental stage-related proteins (**B**) that were identified in the pepper anthers at different developmental stages. (**C**) the X axis represents the different developmental stages, the Y axis represents the number of total identified proteins, and the red bars and blue bars represents the developmental stage-related proteins and other non-developmental stage-related proteins, respectively.

In this study, we identified 1532, 1586, 1603, 1537 and 1446 proteins in the anthers of the pepper CMS line at five developmental stages (Fig. 2A and Supplementary Table S2). Through a comparison of the spectrum intensity of the proteins that were differentially identified by UniProt protein annotation and the Thermo Fisher Sieve 2.2 software, we performed label-free quantitative proteomics and found a total of 136 developmental-stage-related proteins whose expression levels had changed by more than 1.5-fold in at least at one stage (P-value < 0.01). The number of developmental stage-related proteins that were identified in the developmental stages I, II, III, IV and V was 78, 86, 92, 86 and 75, respectively, and the number of developmental stage-related proteins that were identified in only one stage was 15, 16, 7, 5 and 7, respectively. The number of developmental stage-related proteins identified in all five developmental stages was 60, although their expression levels presented significant changes in at least one time period (ratio change >1.5 or <0.67, P-value < 0.01) (Fig. 2B, Table 1 and Supplementary Fig. S1).

Zhang *et al.*^28^ and Wu *et al.*^29^ detected 13 and 23 proteins that were differently expressed between the pepper CMS line and its maintainer line, respectively, by searching databases of green plants and Capsicum. However, we identified more than 1500 proteins by LC MS/MS with the Arabidopsis database. Pepper protein sequence information is limited; however, because Arabidopsis and pepper are both dicotyledonous plants with somewhat homologous features, we used the protein database “Arabidopsis” instead of “Green plants” or “Capsicum.” Although we required accurate data (P-value < 0.01) to perform mass spectrum database identifications, a limited amount of protein data would have caused us to lose important functional protein information. We also required high-throughput proteome identifications to determine the important and meaningful biological results that should be verified in further experiments.

### Gene ontology (GO) annotation function analysis of developmental stage-related proteins

**Figure 3:**
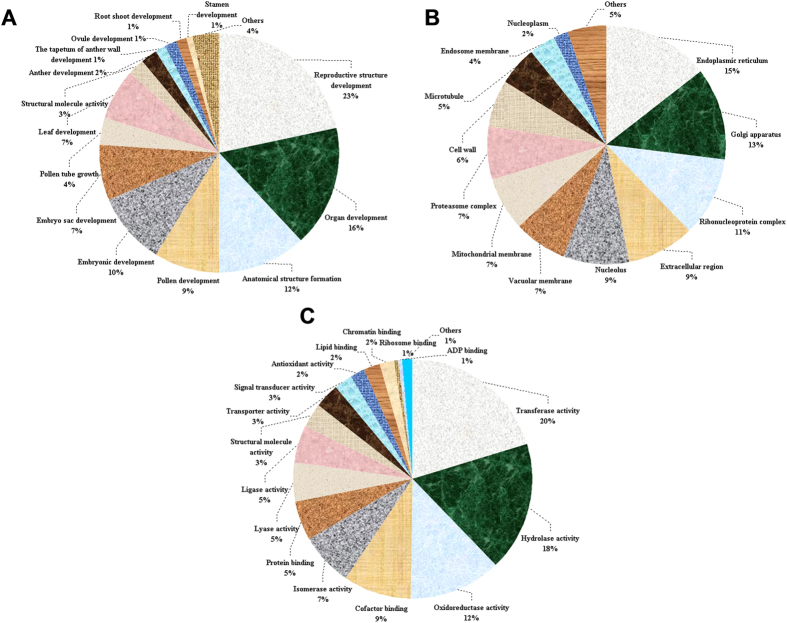
Pie charts classifying the 136 developmental stage-related proteins according to their biological processes (**A**), cellular components (**B**) and molecular functions (**C**). The identified proteins were grouped according to GO annotations and are expressed in percentages.

The 136 developmental stage-related proteins were grouped according to their biological processes, cellular components and molecular functions. Of these proteins, 23% were relevant to genital structure and 9%, 4%, 2% and 1% were directly related to pollen development, pollen tube formation, anther development and tapetal layer changes, respectively (Fig. 3A). Because the CMS line and the maintainer line have the same nuclear background and CMS is maternally inherited, it is widely believed that the main cause of CMS is associated with the inheritance system in the plant cytoplasm^1^. However, we believe that CMS is the result of nucleo-cytoplasmic interactions; thus, the function of the nuclear gene groups in the occurrence of CMS cannot be neglected. In this research, we set the total proteins of anther as the study target without limiting our search cytoplasmic proteins. When we analyzed the cellular components of the developmental stage-related proteins, we found that many differential proteins were enriched in the nuclear protein complexes (11%), whereas the portion of chondriosome proteins was only 7% (Fig. 3B). This result further supports our speculation that nuclear gene groups most likely play a role in the CMS process. Pollen development is relevant to synthesis changes in a large number of proteins, and these synthesis changes often require the synergistic actions of the chondriosome, nuclear protein complexes, endoplasmic reticulum and the Golgi apparatus. Molecular-function clustering was performed for the developmental stage-related proteins, and we found that 20% of the differential proteins had transferase activity and 18% of the enzymes had hydrolase activity (Fig. 3C). The largest identified group was transferases, including acyltransferase, aminotransferase and glycosyl transferase, among others, and it may play an important role in protein post-translational modification (PTM), such as acetylation, amination and glycosylation, during anther development. Thus, the PTM phenomenon is worth studying in future pepper CMS research.

### Possible protein interaction network and metabolic signal pathway changes in anthers of the CMS line

To better understand how anther developmental proteins are interrelated and how proteins that are involved different pathways crosslink to each other, we constructed a PPI network for all of the CMS-related proteins using STRING software. The bioinformatics results indicated that the 136 developmental stage-related proteins whose differential change was greater than 1.5-fold would function in a wide range of biological processes and present different molecular biological functions and would likely have PPI networks. All 136 of the differential responsive proteins constitute a PPI network with pyruvate dehydrogenase (PDH), phosphoglucomutase (PGM) and triosephosphate isomerase (TPI) as its centre (Supplementary Fig. S2), indicating that these proteinases may function synergistically during pollen development in the pepper CMS lines. Among the 136 PPI network proteins, PDH, PGM and TPI were the most prevalent. TPI was also discovered by Wu *et al.*^29^, thus demonstrating its important role in the PPI network of pepper CMS.

We used the KEGG web pathway database to match the identified developmental stage-related proteins and found that many important metabolic key enzymes significantly changed (Supplementary Fig. S3). Thus, we inferred that changes in the amino acid synthesis pathway may be important during anther development in the pepper CMS line. Previous studies have reported that many amino acids, such as proline, asparagine and aspartic acid, occur in different amounts between sterile and fertile plants^45^,^46^,^47^,^48^,^49^,^50^,^51^,^52^, which supports our inference.

### mRNA expression levels of some key enzymes in amino acid synthesis

Based on the proteomics results, we inferred that changes in amino acid synthesis may be important during anther development in the pepper CMS line; therefore, we selected six proteinases (HDH, DAD, ATAAT, CS, P5CS and GS) that are closely related to the amino acid synthesis pathway and investigated their corresponding mRNA expression levels. In addition, it is important to study their transcription levels at different developmental stages.

**Figure 4:**
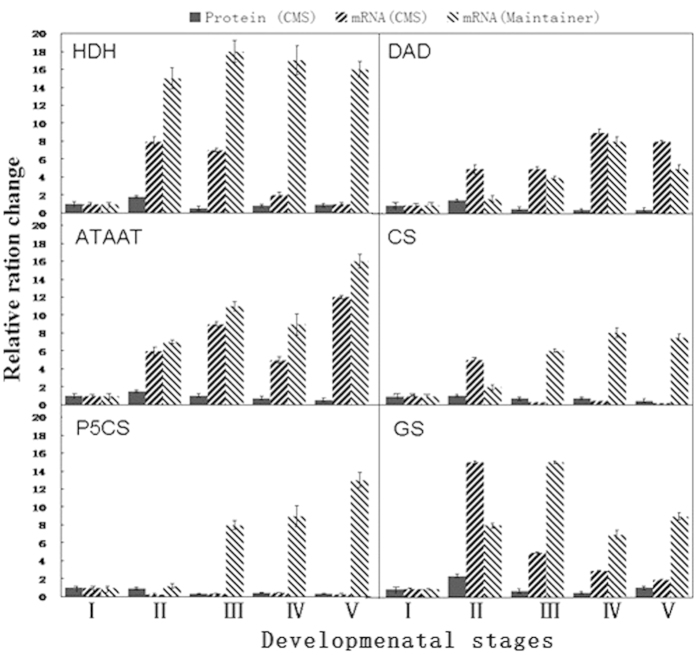
Transcriptional ratio change in HDH, DAD, ATAAT, CS, P5CS and GS at pepper anther developmental stages II, III, IV or V relative to stage I. The gene expression levels were normalized to the expression of the GAPDH gene. Each experiment was repeated three times. The results are shown as mean values. The error bars indicate the s.d. (n = 3).

The results of the experiments indicated that during the different developmental stages of the sterile line anthers, the transcription level changes of HDH, CS, P5CS and GS and the expression level changes of proteins (as identified by quantitative proteomics) were consistent (Fig. 4), whereas the protein and mRNA expression changes of ATAAT and DAD were somewhat inconsistent. Moreover, the protein levels of ATAAT and DAD occasionally decreased with increases in the transcription levels (Fig. 4), which was most likely because some proteins of the corresponding mRNAs were degraded and post-translationally modified after their transcription. A comparison of the mRNA expression levels of the six proteins in the CMS line and maintainer line showed that the transcription levels of some proteins began to decrease at stage II in the sterile line but not in the maintainer line (Fig. 4).

Similar studies have been conducted by Zhang *et al.*^28^, who tested the mRNA expression level of glutathione s-transferase, actin, ketol-acid reductoisomerase, branched-chain α-keto acid dehydrogenase E3 subunit, Cu/Zn superoxide dismutase, aspartic protease and nascent polypeptide-associated complex protein. Only four of these proteins presented a strong correlation between protein abundance and mRNA level, which is similar to our results. This common phenomenon may have been caused by regulation through translation as well as the influence of protein degradation systems on protein expression.

### Western blot analysis of some key enzymes in amino acid synthesis

**Figure 5:**
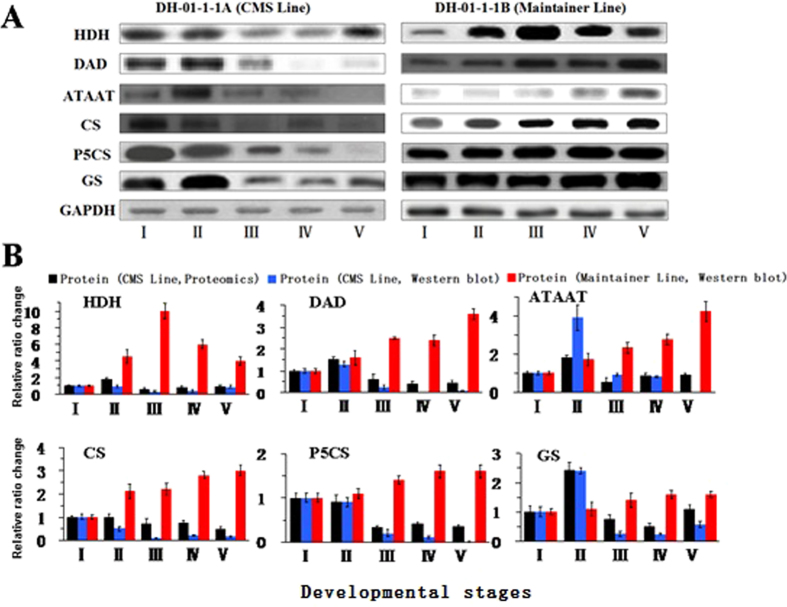
Protein ratio change in HDH, DAD, ATAAT, CS, P5CS and GS at pepper anther developmental stages II, III, IV or V relative to stage I. (**A**) Immunoblots of HDH, DAD, ATAAT, CS, P5CS, GS and GAPDH (control) with their respective antibodies. (**B**) Ratio change for three replicates of the label-free proteomic results and their corresponding immunoblot results. The results are shown as mean values. The error bars indicate the s.d. (n = 3).

To verify the proteomics identification results and determine whether the amino acid synthesis pathway underwent significant changes during anther development in the pepper plants with different fertilities, we prepared polyclone antibodies of the six key enzymes in these processes and performed a western blot analysis in the CMS line and the maintainer line. In the sterile line, the expression levels of HDH, DAD, ATAAT, CS, P5CS and GS significantly decreased at the tapetal layer stage (stage III) (Fig. 5) (the results of the western blot indicated that DAD and P5CS did not exhibit protein-level expression at some developmental stages, which may be why the much higher sensitivity of the mass spectrum facilitates protein expression measurements at the proteome level). However, in the maintainer line anthers, except for the protein expression levels of P5CS and GS, which were roughly smooth, the other key enzymes all showed significantly increased expression at stage II and stage III (Fig. 5). This result indicates that during anther development in the CMS line, the expression levels of many key enzymes in the amino acid synthesis pathways were significantly affected.

### Determination of the content of 17 amino acids in the pepper anthers at different developmental stages

**Figure 6:**
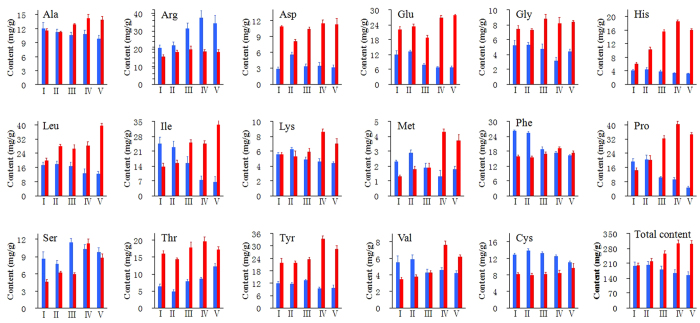
Content of 17 amino acids at five developmental stages (I–V). The blue and red histograms indicate the CMS line and the maintainer line, respectively. The results are shown as mean values. The error bars indicate the s.d. (n = 3).

The content of 17 amino acids during developmental stages I–V in the anthers of the pepper CMS line was analyzed using the amino acid automatic analysis equipment, and the following results were obtained: histidine, valine, cystine, aspartic acid, leucine and isoleucine exhibited a small decrease in the tapetal layer stage (III); proline decreased sharply in the tapetal layer stage (III) and then maintained continuously low expression (Fig. 6 and Supplementary Table S3); serine and tyrosine gradually increased throughout the early stages and then began to decrease in stages IV and V; and the arginine content significantly increased during the later stages, which may have been related to the significant change in proline synthesis. During anther development in the CMS line, the total content of the 17 amino acids slightly increased during the early stages and peaked at stage II, after which it decreased sharply. During stages I–V of the pepper maintainer anthers, except for the content of arginine, which exhibited a slight decrease during the later stages, cystine and aspartic acid all showed smooth expression, whereas histidine, leucine, isoleucine, valine and serine significantly increased and proline rapidly increased, which is in stark contrast to the proline content in the anthers of the CMS line. During anther development in the maintainer line, the total content of the 17 amino acids exhibited a slight increase, peaked at stage IV, and remained steady in stage V (Fig. 6 and Supplementary Table S3). In general, from the tetrad period of meiosis to the tapetal layer stage, the CMS line presents the most significant amino acid changes, which is inconsistent with the results for the maintainer line. The total amino acid content of the anthers of the highly sterile line was significantly lower than that of the maintainer line during the later stages, indicating that the total amino acid synthesis process was significantly affected.

Amino acids are influenced by cytoplasmic sources and other factors^45^. An analysis of the free amino acids in the pepper anthers indicated significant differences in the content of most amino acids between the fertile and CMS lines. In general, our free amino acid data are consistent with those reported by other scientists^45^,^46^,^47^,^48^,^49^. The increased proline content in the free amino acid pool of fertile pepper anthers is common to that of other plant species, and proline deficiencies during the advanced stages of anther development in male sterile versions of various crop plants appear to be common^45^,^46^,^47^,^48^,^49^. Proline may also function as a solute protectant in developmental pollen^48^ and serve to protect the grain from desiccation or other unfavourable environmental conditions during dispersal and prior to germination. Fukasawa *et al.*^46^ reported an accumulation of asparagine and a deficiency of proline and aspartic acid in the anthers of male sterile wheat. Similar amino acid differences were found in our study (Fig. 6). Furthermore, Brooks^50^ found that fertile sorghum anthers have a higher content of aspartic acid, serine and alanine compared with their CMS counterpart, which is similar to our results. This phenomenon has also been observed in Sudan grass^51^ and tomato^52^.

### Significant change in the amino acid synthesis pathway at the anther tapetum stage is essential for pepper cytoplasmic male sterility

Plant fertility is the final result of physiological courses, biochemical reactions and form structuring controlled by a series of genes. Amino acids can function in floral induction as the messengers of the pool of nutrients^53^. Understanding the biochemical mechanisms of male abortion is important when comparing and analyzing differences in the protein and amino acid levels at different developmental stages in both sterile and maintainer lines. The metabolism that occurs inside anthers has a significant impact on pollen fertility. For instance, proline provides nutritive substances, promotes the development and budding of pollen and the elongation of pollen tubes, and it is important because normal pollen contains a significant amount of free proline^54^. Proline is one of the major components of proteins involved in active metabolism and other important functions. Therefore, from the tapetal layer stage on, the proline content in the pepper maintainer line increased significantly (Fig. 6 and Supplementary Table S3), whereas the proline content in the anthers of the sterile line was insufficient and promoted conditions that were not conducive to anther development, thus resulting in microspore abortion. Furthermore, changes in other key enzymes in the amino acid synthesis pathway will also induce metabolic disorders of the pollen grain, affect microspore development and eventually cause male sterility^1^.

**Figure 7:**
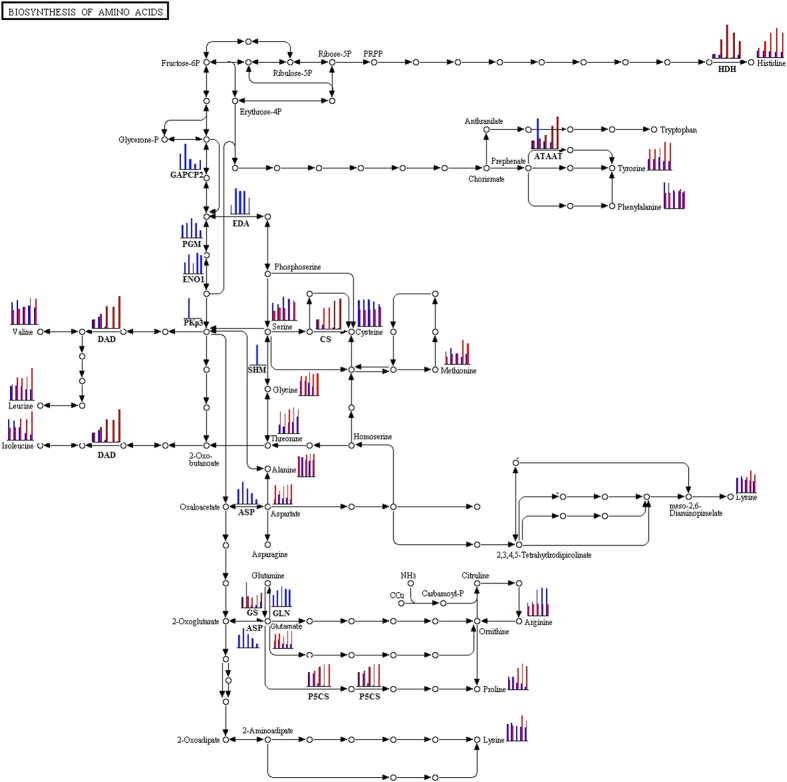
Global view of the change in the amino acid synthesis pathway during the anther maturation in pepper plants with different fertilities. Most of the proteins and amino acids that were quantified by proteomics, immunoblotting and physiological experiments were integrated as blue histograms (CMS line) and red histograms (maintainer line) in the pathway. The protein names are indicated in bold type. As shown in this figure, from the tapetal layer stage on, the amino acid synthesis pathway is severely impeded in the CMS line.

Our experiments indicate that from the tapetal layer stage on, the expression level of most amino acid synthesis-related proteinases sharply decreased and the content of amino acid significantly decreased in the pepper CMS line (Fig. 7). Pepper anthers with different fertilities can be distinguished by determining whether the amino acid synthesis pathways are significantly affected during the anther maturation process, and this process is likely the reason that the tapetal layer develops abnormally and eventually leads to pepper male sterility.

The tapetal layer is the innermost layer of the anther walls, and it has a special secreting function and is indispensable for the development of pollen grains. The proteins that are synthesized in the tapetal layer cells are transported to the anther walls and become extine proteins, which play key roles in the plant’s ability to distinguish pistil stigmas. If the tapetal layer develops abnormally, then male sterility will often occur^55^,^56^,^57^. Combining the results of this experiment with anatomical observations performed during anther development in pepper may lead to discoveries regarding the development of the tapetal layers of the pepper maintainer line, which appear to develop fully from the later stage of the tetrad period and release nutritive substances to the anther cell when the pollen grain has been formed (Fig. 1). During the most important stage of anther development - the pollen grain stage - the total amino acid content along with free proline content in the anther peaks, and these changes play a key role in the formation of pollen (Fig. 6 and Supplementary Table S3). However, the expression levels of the key enzymes in the amino acid synthesis pathways are much higher (Fig. 7). After the meiosis period of the pollen blast cells from the CMS line, vacuolization of the tapetal layer cells begins, and abnormal tapetal layers cannot provide sufficient nutritive substances for pollen formation and development. These abnormal tapetal cells will decompose during the early stage when the microspores are released, which affects the expression levels of key enzymes and causes the accumulation of amino acids and a sharp reduction in the proline content after the development period. Thus, in pepper, male fertility and amino acid synthesis pathway changes are closely related to the development and decomposition of the tapetal layers.

## Conclusions

In this work, we combined cytobiology, proteomics, bioinformatics, immunoblotting and plant physiology to comprehensively investigate the mechanisms underlying pepper CMS. Compared with the anthers of the maintainer line, our results showed that the anthers of the sterile line plants present abnormal tapetal layer cells, and over-vacuolization occurs during anther development; in addition, the expression levels of many key enzymes related to amino acid synthesis decrease sharply at the tapetal layer stage, and the content of many common amino acids significantly decreases at the tapetal layer stage, vacuolization stage and pollen grain crinkling stage. The abnormal development of the CMS line tapetal layers is significant because it affects amino acid synthesis, which may be an important cause of male sterility in pepper.

## Additional Information

**How to cite this article**: Fang, X. *et al.* Involvement of a universal amino acid synthesis impediment in cytoplasmic male sterility in pepper. *Sci. Rep.* **6**, 23357; doi: 10.1038/srep23357 (2016).

## Supplementary Material

Supplementary Information

Supplementary Table S2

## Figures and Tables

**Table 1:** 136 differentially expressed proteins of the pepper anther at different developmental stages.

Uniprot number	Protein names	Score	Unique peptides	Length	pI	Mw/kD	Protein ratio change (Mean ± SD)				
							I	II	III	IV	V
Q9SEI2	26S protease regulatory subunit 6A	738.46	20	424	4.91	47.35	1	1.64 ± 0.21	2.37 ± 0.24	0.95 ± 0.20	0.47 ± 0.11
Q6NQL4	2-dehydro-3-deoxyphosphooctonate aldolase 2	137.69	2	291	5.92	31.54	1	1.22 ± 0.18	1.53 ± 0.12	1.95 ± 0.25	0.94 ± 0.27
P53492	Actin-7	139.41	6	377	5.31	41.74	1	0.80 ± 0.12	0.91 ± 0.13	0.24 ± 0.16	0.25 ± 0.18
Q8L4Y5	Actin-related protein 7	348.54	2	363	4.81	39.90	1	3.20 ± 0.23	2.11 ± 0.32	0.79 ± 0.10	0.52 ± 0.02
Q96329	Acyl-coenzyme A oxidase 4	635.85	22	436	8.60	47.43	1	1.20 ± 0.12	2.15 ± 0.20	2.73 ± 0.16	1.20 ± 0.13
Q9SW96	Asparagine--tRNA ligase	943.90	22	572	5.40	63.65	1	2.40 ± 0.22	0.59 ± 0.14	0.86 ± 0.20	0.58 ± 0.14
Q9FXB0	F25P12.97 protein	432.89	5	166	6.64	18.41	1	0.70 ± 0.09	0.53 ± 0.04	0.50 ± 0.14	1.83 ± 0.20
Q9FYR6	At5g52520 (Prolyl tRNA synthetase)	340.24	2	543	6.55	60.75	1	0.60 ± 0.11	0.19 ± 0.02	1.52 ± 0.21	1.66 ± 0.23
Q9FH02	ATP-dependent zinc metalloprotease FTSH 5	138.59	2	704	5.06	67.21	1	3.21 ± 0.19	4.02 ± 0.51	1.30 ± 0.22	2.33 ± 0.28
Q8W585	ATP-dependent zinc metalloprotease FTSH 8	129.67	2	685	5.17	65.61	1	1.13 ± 0.12	2.53 ± 0.21	1.73 ± 0.16	0.49 ± 0.21
Q9SIE1	Bifunctional aspartate aminotransferase	429.34	5	475	5.97	45.36	1	1.53 ± 0.11	1.09 ± 0.25	0.77 ± 0.12	0.62 ± 0.18
Q94AW8	Chaperone protein dnaJ 3	54.22	2	420	5.76	46.12	1	1.73 ± 0.19	1.82 ± 0.15	0.86 ± 0.24	0.34 ± 0.04
P21238	Chaperonin 60 subunit alpha 1	328.49	3	586	4.80	57.13	1	0.14 ± 0.02	0.80 ± 0.21	1.00 ± 0.23	1.25 ± 0.28
O65902	Cyclase-associated protein 1	137.47	3	476	6.23	50.97	1	1.13 ± 0.14	4.50 ± 0.33	4.36 ± ± 0.44	1.02 ± 0.27
P47998	Cysteine synthase 1	339.92	2	322	5.92	33.67	1	1.73 ± 0.16	0.80 ± 0.14	0.82 ± 0.13	0.52 ± 0.07
P47999	Cysteine synthase, chloroplastic/chromoplastic	146.28	2	392	5.54	35.09	1	1.02 ± 0.17	0.72 ± 0.09	0.75 ± 0.11	0.48 ± 0.09
O49485	D-3-phosphoglycerate dehydrogenase 1	149.01	2	603	5.52	57.87	1	3.11 ± 0.24	1.64 ± 0.27	1.76 ± 0.25	1.85 ± 0.22
P54887	Delta-1-pyrroline-5-carboxylate synthase A	98.41	2	717	5.89	77.70	1	0.91 ± 0.11	0.33 ± 0.09	0.42 ± 0.03	0.36 ± 0.03
Q9LIR4	Dihydroxy-acid dehydratase	109.82	2	608	5.44	61.06	1	1.53 ± 0.33	0.62 ± 0.08	0.42 ± 0.05	0.44 ± 0.04
Q9SI75	Elongation factor G	430.06	4	783	5.06	77.67	1	3.24 ± 0.12	1.35 ± 0.23	1.84 ± 0.20	2.60 ± 0.15
Q9C9C4	Enolase 1	125.74	2	477	5.15	46.81	1	1.67 ± 0.13	0.94 ± 0.20	1.85 ± 0.23	1.62 ± 0.26
Q9XI91	Eukaryotic translation initiation factor 5A-1	118.16	2	158	5.41	17.36	1	1.47 ± 0.21	3.22 ± 0.22	2.24 ± 0.20	2.74 ± 0.29
Q8W104	F-box/LRR-repeat protein 17	155.46	2	593	8.31	64.77	1	0.94 ± 0.10	0.47 ± 0.07	0.25 ± 0.05	0.30 ± 0.06
P93033	Fumarate hydratase 1	227.03	2	492	6.64	49.94	1	1.32 ± 0.16	2.30 ± 0.27	2.52 ± 0.28	1.02 ± 0.20
P55228	Glucose-1-phosphate adenylyltransferase small subunit	124.32	2	520	5.33	49.36	1	2.94 ± 0.28	2.21 ± 0.21	1.80 ± 0.23	1.27 ± 0.27
Q43127	Glutamine synthetase	751.98	20	430	5.28	42.47	1	1.44 ± 0.33	1.78 ± 0.09	1.53 ± 0.05	1.09 ± 0.24
Q9LV03	Glutamate synthase 1 [NADH]	109.41	2	2208	5.80	236.71	1	1.55 ± 0.20	0.67 ± 0.08	0.86 ± 0.13	0.53 ± 0.11
P25858	Glyceraldehyde-3-phosphate dehydrogenase GAPC1	74.22	8	338	6.62	36.91	1	3.21 ± 0.28	1.54 ± 0.23	1.34 ± 0.20	0.39 ± 0.04
Q5E924	Glyceraldehyde-3-phosphate dehydrogenase GAPCP2	534.22	2	420	6.37	37.95	1	1.65 ± 0.14	0.68 ± 0.07	0.36 ± 0.06	0.58 ± 0.08
Q03250	Glycine-rich RNA-binding protein 7	109.51	2	176	5.89	16.76	1	0.68 ± 0.02	0.94 ± 0.15	0.24 ± 0.03	0.35 ± 0.05
Q9C4Z6	Guanine nucleotide-binding protein subunit beta-like protein B	146.44	2	326	6.66	35.80	1	1.35 ± 0.17	1.67 ± 0.22	0.78 ± 0.08	0.88 ± 0.21
Q9C5U8	Histidinol dehydrogenase (HDH)	143.07	3	466	5.34	47.05	1	1.83 ± 0.12	0.53 ± 0.22	0.86 ± 0.11	0.90 ± 0.10
Q944P7	Leucine aminopeptidase 3	132.21	2	583	5.66	53.67	1	0.98 ± 0.11	1.58 ± 0.26	1.99 ± 0.27	2.15 ± 0.25
Q9SIT1	Leucine-rich repeat receptor-like protein kinase	162.50	2	943	6.35	63.23	1	3.21 ± 0.19	0.68 ± 0.07	0.48 ± 0.09	0.98 ± 0.21
Q9LXL8	Maternal effect embryo arrest 38 protein	95.57	2	295	9.09	33.36	1	1.35 ± 0.08	1.68 ± 0.25	0.47 ± 0.06	0.67 ± 0.08
Q9LKR3	Mediator of RNA polymerase II transcription subunit 37a (Heat shock 70 kDa protein 11)	134.22	7	669	5.05	70.85	1	2.20 ± 0.29	3.20 ± 0.26	3.40 ± 0.23	1.90 ± 0.24
Q39024	Mitogen-activated protein kinase 4 (AtMPK4)	88.93	6	376	5.74	42.85	1	0.71 ± 0.08	1.74 ± 0.24	1.91 ± 0.25	0.43 ± 0.04
Q9C566	Peptidyl-prolyl cis-trans isomerase CYP40 (PPIase CYP40)	131.55	3	361	5.61	40.61	1	1.49 ± 0.15	1.84 ± 0.22	0.86 ± 0.20	0.97 ± 0.29
Q9ZPI6	Peroxisomal fatty acid beta-oxidation multifunctional protein AIM1	132.10	6	721	9.36	77.86	1	1.68 ± 0.09	1.35 ± 0.15	1.72 ± 0.18	1.64 ± 0.22
Q9M9K1	Probable 2,3-bisphosphoglycerate-independent phosphoglycerate mutase 2	71.23	4	560	5.53	60.63	1	1.18 ± 0.06	1.54 ± 0.21	1.19 ± 0.22	0.67 ± 0.08
Q9SJ12	Probable ATP synthase 24 kDa subunit	152.53	2	240	5.30	24.00	1	3.53 ± 0.45	0.75 ± 0.07	0.53 ± 0.05	1.08 ± 0.20
Q9LX12	Probable inositol 3-phosphate synthase isozyme 3	119.93	5	510	5.58	56.42	1	1.49 ± 0.10	1.85 ± 0.17	0.52 ± 0.05	0.74 ± 0.11
Q9M8Y0	Probable UDP-N-acetylglucosamine--peptide N-acetylglucosaminyltransferase SEC	122.42	2	977	6.85	110.11	1	2.42 ± 0.43	3.52 ± 0.45	3.74 ± 0.36	2.09 ± 0.34
Q9M9P3	Probable UTP--glucose-1-phosphate uridylyltransferase 2	142.22	7	469	5.80	51.61	1	0.77 ± 0.09	1.87 ± 0.28	2.09 ± 0.36	0.44 ± 0.08
Q39224	Protein SRG1	734.22	20	358	5.30	41.04	1	1.63 ± 0.05	2.02 ± 0.20	0.94 ± 0.21	1.06 ± 0.21
P31414	Pyrophosphate-energized vacuolar membrane proton pump 1	118.14	2	770	5.13	80.82	1	1.85 ± 0.14	1.49 ± 0.17	1.89 ± 0.22	1.80 ± 0.26
F4JGR5	Pyrophosphate--fructose 6-phosphate 1-phosphotransferase subunit beta 2	120.38	2	569	5.44	62.74	1	1.19 ± 0.18	1.91 ± 0.28	2.41 ± 0.27	2.60 ± 0.28
Q9C958	Serine/threonine-protein kinase SRK2B	131.56	4	361	5.62	41.04	1	3.88 ± 0.47	0.82 ± 0.12	0.58 ± 0.08	1.19 ± 0.21
Q00917	Sucrose synthase 2	118.26	2	807	5.70	92.06	1	1.63 ± 0.09	2.03 ± 0.18	0.57 ± 0.06	0.81 ± 0.09
Q9M111	Sucrose synthase 3	138.21	6	809	5.85	92.00	1	2.66 ± 0.16	3.87 ± 0.22	4.11 ± 0.54	2.30 ± 0.26
Q39243	Thioredoxin reductase 1	140.74	2	375	6.96	39.63	1	0.85 ± 0.10	2.06 ± 0.26	2.30 ± 0.27	0.48 ± 0.12
Q9FLE9	Transcription factor PRE1	140.40	2	92	9.09	10.51	1	1.93 ± 0.12	0.92 ± 0.15	0.80 ± 0.08	0.23 ± 0.04
P31265	Translationally-controlled tumor protein	65.32	2	168	4.52	18.91	1	0.39 ± 0.03	0.41 ± 0.11	0.82 ± 0.14	0.95 ± 0.16
Q9SKP6	Triosephosphate isomerase	119.62	2	315	5.38	27.05	1	1.11 ± 0.17	0.56 ± 0.12	0.54 ± 0.14	0.21 ± 0.02
Q9LFW1	UDP-arabinopyranose mutase 2	123.87	3	360	5.77	40.76	1	0.81 ± 0.11	1.01 ± 0.21	0.47 ± 0.12	0.53 ± 0.15
P57751	UTP--glucose-1-phosphate uridylyltransferase 1	336.83	3	470	5.73	51.79	1	0.92 ± 0.13	0.74 ± 0.12	0.94 ± 0.17	0.89 ± 0.16
O23654	V-type proton ATPase catalytic subunit A	142.90	3	623	5.11	68.81	1	0.59 ± 0.07	0.95 ± 0.17	1.19 ± 0.11	1.29 ± 0.16
Q9SDS7	V-type proton ATPase subunit C	70.25	2	375	5.40	42.62	1	1.93 ± 0.23	0.41 ± 0.04	0.29 ± 0.01	0.59 ± 0.09
Q39258	V-type proton ATPase subunit E1	140.59	2	230	6.04	26.06	1	0.81 ± 0.09	1.01 ± 0.17	0.28 ± 0.03	0.40 ± 0.08
O22607	WD-40 repeat-containing protein MSI4	135.03	2	507	5.81	55.76	1	1.32 ± 0.21	1.92 ± 0.22	2.04 ± 0.25	1.14 ± 0.19
Q9SZI2	Nucleosome assembly protein 1	116.14	2	372	4.36	42.61	1	–	1.72 ± 0.14	–	1.08 ± 0.09
P46644	Aspartate aminotransferase 3	77.81	2	449	9.30	44.49	1	1.08 ± 0.16	–	0.81 ± 0.13	0.72 ± 0.08
Q9FHW7	SKP1-like protein 1B	96.92	2	171	4.51	19.10	1	–	1.44 ± 0.13	1.44 ± 0.23	1.35 ± 0.12
Q42553	Isopentenyl-diphosphate Delta-isomerase II	122.05	2	284	5.16	27.53	–	1	2.35 ± 0.12	2.96 ± 0.28	–
Q39102	ATP-dependent zinc metalloprotease FTSH 1	129.73	3	716	5.20	67.62	–	1	3.40 ± 0.34	0.95 ± 0.10	–
Q39043	Mediator of RNA polymerase II transcription subunit 37f	122.45	5	668	5.08	71.14	–	1	6.48 ± 0.86	6.89 ± 0.59	–
O80763	Probable nucleoredoxin 1	119.87	3	578	4.90	65.17	–	1	3.44 ± 0.43	3.85 ± 0.25	–
Q38882	Phospholipase D alpha 1	111.44	4	810	5.53	91.85	–	1	3.72±0.33	1.74 ± 0.24	–
Q8LPS1	Long chain acyl-CoA synthetase 6	121.81	2	701	6.97	72.60	–	1	2.74 ± 0.26	–	–
Q8H1B3	Probable mediator of RNA polymerase II transcription subunit 37b	110.28	2	675	4.89	71.26	–	1	3.52 ± 0.41	–	–
Q9C5I1	UDP-sugar pyrophosphorylase	136.52	6	614	6.07	67.72	–	1	5.10 ± 0.61	–	–
Q9ZNT0	Protein SUPPRESSOR OF K(+) TRANSPORT GROWTH DEFECT 1	125.73	3	435	6.50	48.59	–	1	4.05 ± 0.53	–	–
Q96293	Actin-8	127.56	6	377	5.37	41.86	–	–	1	0.90 ± 0.15	–
Q8LK56	Transcriptional activator DEMETER	121.89	2	1987	7.61	221.15	–	–	1	1.94 ± 0.20	–
O24456	Guanine nucleotide-binding protein subunit beta-like protein A	116.76	3	327	7.61	35.75	–	–	1	1.90 ± 0.18	–
Q93W54	Protein-S-isoprenylcysteine O-methyltransferase B	116.80	5	197	9.24	22.77	–	–	1	2.38 ± 0.28	–
O04331	Prohibitin-3, mitochondrial	111.37	2	277	7.18	30.27	–	–	1	0.22 ± 0.08	–
P10896	Ribulose bisphosphate carboxylase/oxygenase activase	114.01	3	474	5.09	46.26	–	–	1	0.28 ± 0.02	–
P49227	60S ribosomal protein L5-2	121.92	2	301	9.18	34.44	–	–	1	0.57 ± 0.04	–
O23255	Adenosylhomocysteinase 1	107.56	6	485	5.66	53.38	–	–	1	1.14 ± 0.20	–
P12411	Tubulin beta-1 chain (Beta-1-tubulin)	121.04	2	447	4.68	50.22	–	–	1	0.24 ± 0.05	–
O22263	Protein disulfide-isomerase like 2-1	117.68	3	361	5.65	37.17	–	–	1	0.54 ± 0.04	0.30 ± 0.05
F4I6W4	Phosphoglucomutase	117.65	2	662	6.67	72.56	–	–	1	1.38 ± 0.12	0.72 ± 0.11
O64688	Pyruvate dehydrogenase E1 component subunit beta-3	112.11	2	406	5.03	36.81	–	–	1	0.36 ± 0.06	0.48 ± 0.05
Q9M8Z7	Sterol 3-beta-glucosyltransferase UGT80A2	91.22	2	637	6.27	69.28	–	–	1	0.30 ± 0.04	0.78 ± 0.05
Q9FHH2	101 kDa heat shock protein; HSP101-like protein	116.21	3	990	8.32	108.71	–	–	1	0.72 ± 0.06	0.96 ± 0.17
Q9SAK5	Myb family transcription factor APL	113.50	3	358	8.73	39.60	*	–	–	–	–
Q8VYD9	CRS2-associated factor 1	267.21	3	405	9.24	43.97	*	–	–	–	–
Q67XD9	Alkaline/neutral invertase CINV2	116.19	3	558	6.11	64.23	*	–	–	–	–
Q43125	Cryptochrome-1 (Blue light photoreceptor)	114.83	5	681	5.23	76.69	*	–	–	–	–
Q9FKI0	Fimbrin-5	119.82	3	687	5.13	76.76	*	–	–	–	–
Q9SAJ6	Glyceraldehyde-3-phosphate dehydrogenase GAPCP1	355.68	4	422	6.79	36.89	*	–	–	–	–
Q56WN1	Glutamine synthetase cytosolic isozyme 1-1	84.22	2	356	5.28	38.98	*	–	–	–	–
Q9FX31	Homeobox-leucine zipper protein HDG11	129.26	3	722	6.23	79.12	*	–	–	–	–
Q9FNX8	Lipoxygenase 4	224.21	4	926	6.37	98.30	*	–	–	–	–
Q9SMU8	Peroxidase 34	128.49	2	353	7.71	35.70	*	–	–	–	–
Q9SZD4	26S proteasome regulatory subunit 4 homolog A	120.93	2	443	5.92	49.37	*	–	–	–	–
Q9LNC5	Elongation factor like protein	121.56	2	987	5.05	110.44	*	–	–	–	–
Q9SKN5	Auxin response factor 10	131.45	2	693	7.63	76.72	*	–	–	–	–
Q9SP02	Peptidyl-prolyl cis-trans isomerase CYP20-1	117.32	2	204	9.08	19.58	*	–	–	–	–
Q9LYD9	Protein EMBRYONIC FLOWER 1	120.49	3	1096	9.35	121.67	*	–	–	–	–
Q39101	Ferritin-1, chloroplastic	329.74	4	255	5.19	23.53	–	–	–	–	–
Q00958	Protein LEAFY	118.55	2	420	6.65	46.58	–	–	–	–	–
Q9C6Z1	Probable 9-cis-epoxycarotenoid dioxygenase NCED5	122.45	2	589	5.35	60.46	–	*	–	–	–
Q93Z53	Plastidial pyruvate kinase 3	67.11	2	571	6.41	56.87	–	*	–	–	–
Q9C888	Phospholipase D epsilon	112.66	2	762	7.53	86.77	–	*	–	–	–
O04019	26S protease regulatory subunit 6A homolog B	610.29	3	423	4.90	47.04	–	*	–	–	–
Q9SRT9	UDP-arabinopyranose mutase 1	127.35	2	357	5.61	40.50	–	*	–	–	–
Q0WV90	Topless-related protein 1	126.87	2	1120	6.68	124.09	–	*	–	–	–
Q8LAC4	Probable uridine nucleosidase 2	115.64	2	322	5.02	34.67	–	*	–	–	–
Q9SA69	BTB/POZ domain-containing protein At1g03010	72.01	4	634	8.87	71.16	–	*	–	–	–
Q9LF88	Late embryogenesis abundant protein-like	123.98	2	479	5.29	52.08	–	*	–	–	–
Q9ZVW2	Expressed protein (Putative DEAD/DEAH box RNA helicase (HUA ENHANCER2))	65.56	2	995	6.02	111.89	–	*	–	–	–
Q9LJE2	Lysine--tRNA ligase	112.35	2	602	5.73	67.59	–	*	–	–	–
P35632	Floral homeotic protein APETALA 3	109.01	4	232	8.71	27.34	–	*	–	–	–
Q9M086	DDB1- and CUL4-associated factor homolog 1	85.84	2	1883	4.98	205.45	–	*	–	–	–
Q9ZSM8	Histone-lysine N-methyltransferase	125.38	2	856	6.14	95.40	–	*	–	–	–
Q9LYN2	Ferritin-3, chloroplastic	121.27	3	259	5.13	23.76	–	-	*	–	–
Q9SVM4	Serine hydroxymethyltransferase 5	76.48	2	470	5.66	52.26	–	–	*	–	–
Q9C9P4	3-oxoacyl-[acyl-carrier-protein] synthase II	67.26	2	541	6.09	46.76	–	–	*	–	–
P24101	Peroxidase 33	126.61	3	354	7.04	35.62	–	–	*	–	–
Q9FF53	Probable aquaporin PIP2-4	226.80	2	291	8.22	30.95	–	–	*	–	–
Q9SL67	26S proteasome regulatory subunit 4 homolog B	114.79	2	443	5.85	49.22	–	–	*	–	–
P49209	60S ribosomal protein L9-1	99.11	2	194	9.48	22.02	–	–	*	–	–
Q6Q4D0	Protein TONSOKU (Protein BRUSHY 1)	114.14	2	1311	5.45	146.48	–	–	–	*	–
Q94A97	Ubiquitin-conjugating enzyme E2 35	125.68	2	153	6.74	17.19	–	–	–	*	–
F4JYE9	Folylpolyglutamate synthase	115.98	3	530	6.72	56.90	–	–	–	*	–
F4KF14	AAA-type ATPase family protein	68.84	2	855	5.67	96.85	–	–	–	*	–
O22161	Protein ARABIDILLO 1	320.40	2	930	5.97	100.64	–	–	–	*	–
Q9SRT3	Probable xyloglucan glycosyltransferase 6	127.40	2	682	8.99	78.37	–	–	–	–	*
Q9STX5	Endoplasmin homolog	108.24	2	823	4.90	91.53	–	–	–	–	*
Q67Z93	Inactive protein FRIGIDA	316.84	2	314	8.57	34.97	–	–	–	–	*
Q9SHJ5	Glycerol-3-phosphate acyltransferase 1	115.19	2	585	9.24	66.51	–	–	–	–	*
Q9LJM4	Receptor-like protein kinase HAIKU2	108.70	2	991	7.26	108.26	–	–	–	–	*
Q8S905	Kinesin-like protein NACK1	69.32	2	974	7.87	110.17	–	–	–	–	*
Q8L7L0	GTP-binding protein OBGC	70.32	3	681	4.95	72.08	–	–	–	–	*

*indicates that the protein was only identified at the corresponding developmental stage.

-indicates that no protein was only identified at the corresponding developmental stage.
