# Supplementary material for: Involvement of a universal amino acid synthesis impediment in cytoplasmic male sterility in pepper
Source: Sci Rep. 2016 Mar 18;6:23357. doi: 10.1038/srep23357 (PMC4796900; doi:10.1038/srep23357)
Supplement: Supplementary Information [file srep23357-s1.pdf]

Involvement of a universal amino acid synthesis impediment in cytoplasmic male sterility in pepper

Xianping Fang[1], Hong-Fei Fu[2], Zhen-Hui Gong[3] and Wei-Guo Chai[2,*]

[1]Institute of Biology, Hangzhou Academy of Agricultural Sciences, Hangzhou 310024, China

[2]Institute of Vegetables, Hangzhou Academy of Agricultural Sciences, Hangzhou 310024, China

[3]College of Horticulture, Northwest A&F University, Yangling 712100, China

Xianping Fang: fxpbio@163.com

Hong-Fei Fu: 10612339@qq.com

Zhen-Hui Gong: zhgong@nwsuaf.edu.cn

*Corresponding author:

Wei-Guo Chai: Tel, +86-571-86299805; Fax, +86-571-86299805; E-mail, kuni@21cn.com

Supplementary Figures

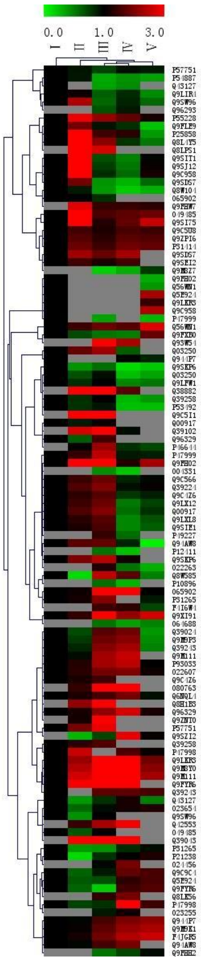

Figure S1 Clustering chart of 136 identified responsive proteins of pepper anther CMS line at five developmental stages based on expression level

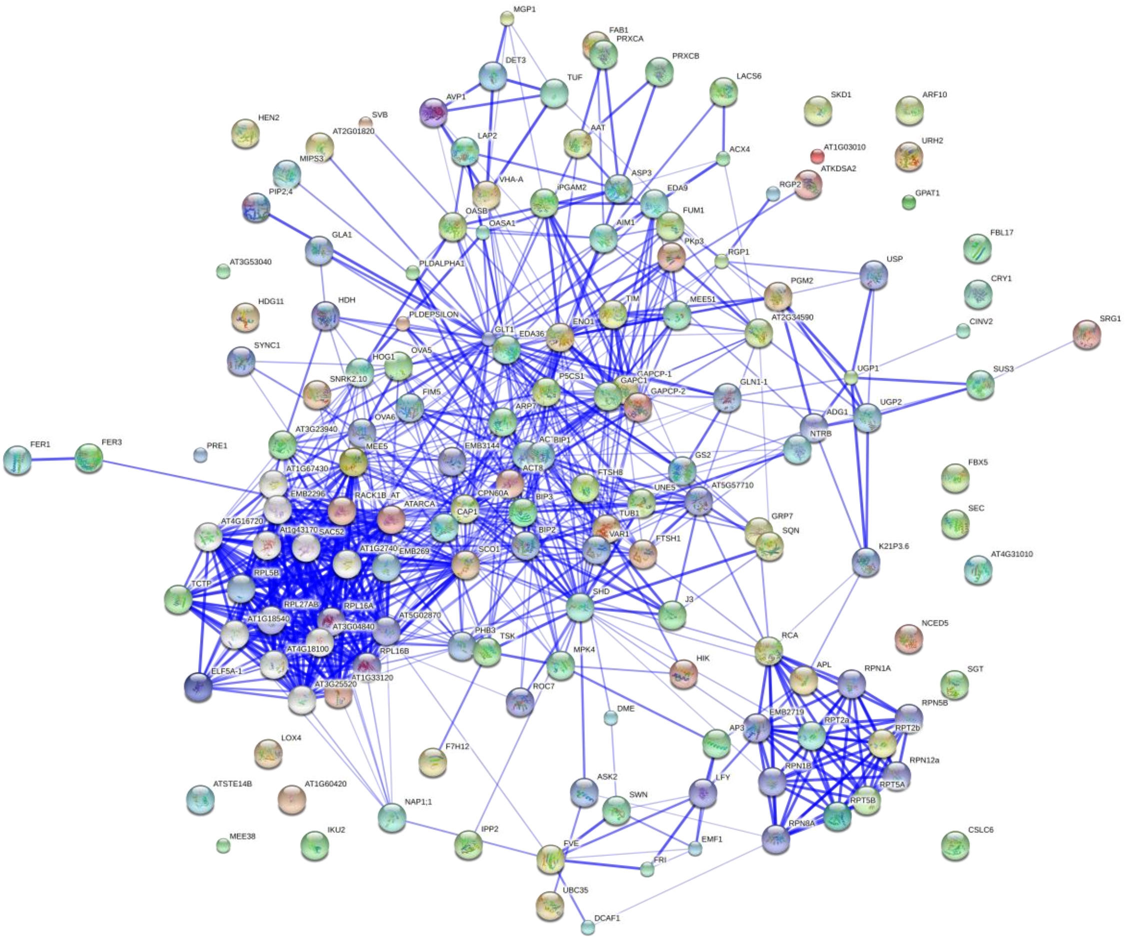

Figure S2 Protein-protein interaction network of 136 differential expressed proteins

This is the confidence view of String software. Stronger associations are represented by thicker lines.136 proteins are found in the network involving in the anther CMS developmental process of pepper.

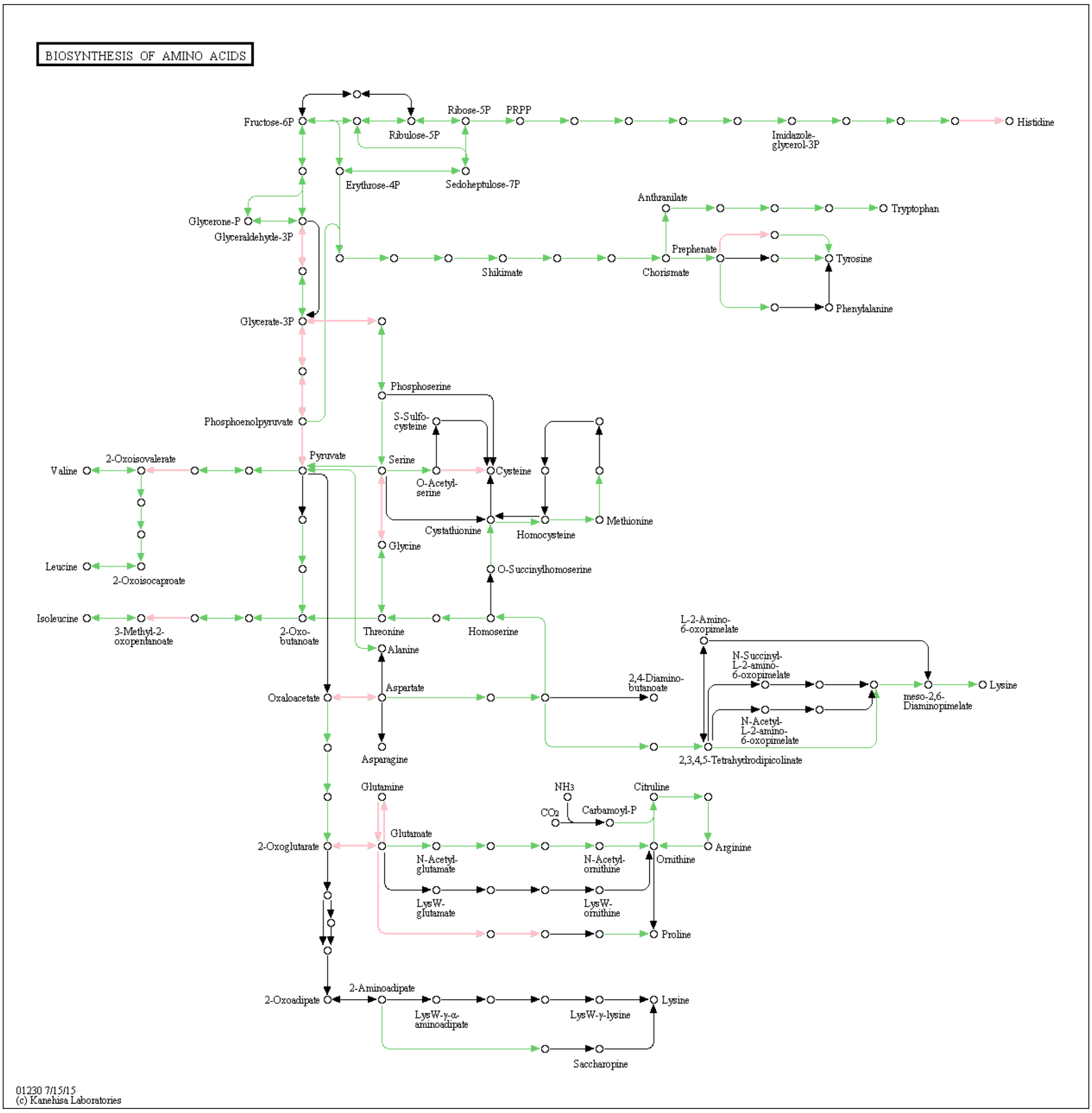

Figure S3 Proteins involving in the synthesis pathway of amino acids

Each pink line indicates a corresponding identified protein by the label-free proteomics method.

Supplementary Tables

Table S1 Primers of six corresponding genes for RT-PCR

**Corresponding proteins (Uniprot number)**	**Primer**	**Sequence（5’-3’）**	**Tm/℃**	**Usage**
HDH (Q9C5U8)	GDP-S<br>GDP-A	GGCCTGGGCCACGttnckngccat<br>TGCTCCTACAACATGACCccngargaryt	61.2	Homologous cloning
	5’GSP	AGGACACATGTGGGCATGGGT	70.1	5’RACE
	3’GSP	TGGCACGTCGTCGCGCTCGCT	71.8	3’RACE
	GFP-S<br>GFP-A	GCAGCCCGGGCAGGTTCACG<br>GACACATGTGGGCATGGGTGCAA	56.8	Full validation
	GRTP-S<br>GRTP-A	AGCCCGGGCAGGTTCACGAT<br>TGTGGGCATGGGTGCAAAATGT	57.1	RT-PCR
DAD (Q9LIR4)	GDP-S<br>GDP-A	GCTGAACTCCGACGGCttygargtnat<br>CGGAGGACACCAGGATCacytcraancc	61.0	Homologous cloning
	5’GSP	GGTGCGGTTGGTCTTGGCAGGCA	70.1	5’RACE

	3’ GSP	TCGACCCCGACTGGACCACGAGC	70.3	3’RACE
	GFP-S	GGTTGGGACAGCAGTTGTTA	57.8	Full validation
	GFP-A	ATGTGTAGGTAGCTTACAATGACA		
	GRTP-S	GACGACGACGATAATGGAGG	57.6	RT-PCR
	GRTP-A	TGTAGGTAGCTTACAATGACAAGA		
ATAAT (Q9SIE1)	GDP-S	CGCAGCACGAGCTGgayggnaargc	62.2	Homologous cloning
	GDP-A	CCAGCTGGTCGAACATGtytcrgatrat		
	5’ GSP	TGGTGCGGTTGGTCTTGGCAGGCA	71.2	5’RACE
	3’ GSP	CGACCCCGACTGGACCACGAGCA	70.4	3’RACE
	GFP-S	TCAATAGCAGCTTTGCGGAT	57.6	Full validation
	GFP-A	GTGTAGGTAGCTTACAATGACAAG		
	GRTP-S	GACGACGATAATGGAGGAGC	56.2	RT-PCR
	GRTP-A	GTGTAGGTAGCTTACAATGACAA		
CS (P47999)	GDP-S	GTTCCGGAAGCAGCTGaaygaracngt	60.1	Homologous cloning
	GDP-A	AGGTCCAGCATGGACTTCacngtyttcrt		
	5’ GSP	TCTGGTGCGGTTGGTCTTGGCAGGC	71.3	5’RACE
	3’ GSP	CTGCCCGTGCTTACCCCCACCTCT	70.2	3’RACE
	GFP-S	CGTTCCTCTCTCTGTGTGTG	57.0	Full validation

	GFP-A	ATGTGTAGGTAGCTTACAATGAC		
	GRTP-S	TCGTTCACGTACTTTTGCCA	57.2	RT-PCR
	GRTP-A	GATCAGAAATGTGTAGGTAGCTT		
P5CS (P54887)	GDP-S	TCCGGCATCTTCtgggayaayga	61.2	Homologous cloning
	GDP-A	GACAGCAGGATCAGCAGGTcngcyttnaryt		
	5’ GSP	CTGGTGCGGTTGGTCTTGGCAGGC	70.4	5’RACE
	3’ GSP	TTCGACCCCGACTGGACCACGAGC	70.2	3’RACE
	GFP-S	ACTTGATGGGAAGGCTTGTG	57.1	Full validation
	GFP-A	AAGATCAGAAATGTGTAGGTAGC		
	GRTP-S	CCATAGGGTTTGTGAGTTTGC	58.5	RT-PCR
	GRTP-A	ACCCGGCAAGTAAGACACAA		
GS (Q9LV03)	GDP-S	CGGCTACTCCGCCgaraayathga	60.3	Homologous cloning
	GDP-A	ACTCCCGGGCGGcnacngccatrt		
	5’ GSP	CTGGTGCGGTTGGTCTTGGCAGGCA	71.5	5’RACE
	3’ GSP	GGATCCTGCCCGTGCTTACCCCCA	70.3	3’RACE
	GFP-S	ATGTACCCTCAACCTTAAAACCC	59.3	Full validation
	GFP-A	CCCGCGGCAATCCAAAGATA		
	GRTP-S	GTACCCTCAACCTTAAAACCCT	58.5	RT-PCR
	GRTP-A	GATGCCCGCGACAATTCAAC		
GAPDH (gene symbol AJ246013) (Reference)	GRTP-S	GAAGAATTGGTCGATTGGTG	58.0	Internal control
	GRTP-A	CCCCGTTGACTCTACGACAT		Internal control

Table S3 Content of 17 amino acids at five developmental stages

Amino acids	Content of amino acids at five developmental stages（mg/g）									
	DH - 0 1 - 1 - 1 A (CMS Line)					DH - 0 1 - 1 - 1 B (Maintainer Line)				
	I	II	III	Ⅳ	V	I	II	III	Ⅳ	V
Alanine	12.1±2.3	11.3±1.5	10.6±1.3	10.9±2.0	9.9±1.2	11.6±1.0	11.2±0.9	12.9±0.6	14.3±0.4	13.9±1.2
Arginine	20.6±1.6	22.0±1.9	31.6±2.8	37.5±3.9	34.3±4.7	15.6±1.2	18.3±1.1	19.6±2.0	18.5±1.2	18.3±1.4
Aspartic acid	2.9±0.3	5.6±0.4	3.4±0.4	3.5±0.6	3.2±0.4	10.9±0.2	8.1±0.3	10.4±0.4	11.5±0.6	11.3±1.1
Glutamic acid	12.0±1.5	13.3±0.5	7.8±0.6	6.9±0.2	6.8±0.7	22.0±1.2	23.3±0.9	18.8±1.0	26.9±0.8	27.8±0.5
Glycine	5.2±0.7	5.3±0.3	4.8±0.6	3.2±0.4	4.4±0.3	7.4±0.5	7.3±0.2	8.8±0.6	8.2±0.8	8.4±0.2
Histidine	4.1±0.2	4.4±0.6	3.8±0.4	3.3±0.2	3.2±0.1	6.1±0.4	10.4±0.5	15.7±0.4	18.7±0.3	16.0±0.5
Leucine	17.3±1.8	17.9±1.7	16.8±2.1	12.9±2.2	12.6±1.2	20.1±1.2	27.9±1.1	26.8±2.0	28.3±2.2	39.6±1.3
Isoleucine	24.6±3.1	22.9±2.6	15.3±3.1	7.6±1.6	6.5±2.4	13.8±1.5	15.3±1.4	25.0±1.5	24.6±1.2	33.5±2.0
Lysine	5.6±0.2	6.3±0.2	4.9±0.3	4.6±0.4	4.4±0.2	5.6±0.2	5.3±0.7	5.9±0.5	8.6±0.4	7.0±0.7
Methionine	2.3±0.1	2.9±0.2	1.9±0.3	1.3±0.4	1.8±0.2	1.3±0.1	1.8±0.2	1.9±0.3	4.3±0.2	3.7±0.4
Phenylalanine	26.3±0.3	25.4±0.6	18.9±0.8	17.4±0.5	16.4±0.3	16.0±0.4	15.5±0.6	16.9±0.8	19.2±0.4	17.4±0.7
Proline	19.5±1.3	20.6±2.4	10.4±0.4	9.4±0.8	4.7±0.19	14.5±1.2	20.4±2.2	32.4±1.4	40.4±1.8	34.7±1.1
Serine	8.6±1.2	7.7±0.6	11.5±0.6	10.3±0.8	9.8±0.7	4.6±0.4	6.2±0.2	6.0±0.2	11.3±0.7	8.8±0.7
Threonine	6.3±0.8	4.9±0.6	7.8±0.6	8.6±0.4	12.3±0.8	16.0±0.9	14.4±0.3	17.8±1.6	19.6±1.4	12.3±0.8
Tyrosine	11.9±0.8	11.7±0.5	13.5±0.4	9.4±0.7	9.7±1.3	21.9±2.0	21.7±1.0	23.5±0.9	33.4±1.3	28.5±1.7
Valine	5.5±0.8	5.9±0.5	4.3±0.4	4.6±0.3	4.2±0.3	3.5±0.2	3.8±0.2	4.3±0.2	7.6±0.5	6.2±0.2
Cystine	12.9±0.3	13.9±0.4	13.3±0.3	12.5±0.4	11.0±0.09	8.2±0.3	7.9±0.5	8.2±0.4	8.5±0.6	9.7±0.1
Total content	197.7±17.3	202.0±15.5	180.6±15.4	163.9±15.8	155.2±15.1	199.1±12.9	218.8±12.3	254.9±14.8	303.9±14.8	302.1±14.6
